# Complete mitochondrial genome and phylogenetic analysis of the marine microalga *Symbiochlorum hainanensis* (Ulvophyceae, Chlorophyta)

**DOI:** 10.1080/23802359.2023.2290353

**Published:** 2023-12-18

**Authors:** Fangfang Yang, Lijuan Long

**Affiliations:** Key Laboratory of Tropical Marine Bio-resources and Ecology, South China Sea Institute of Oceanology, Chinese Academy of Sciences, Guangzhou, China

**Keywords:** *Symbiochlorium hainandiae*, mitochondrial genome, phylogenetic analysis

## Abstract

*Symbiochlorum hainanensis* Gong et al. (2018). is a unicellular green alga belonging to Ulvophyceae, Chlorophyta, and considered as an important species in coral-algae symbiont areas. In this study, we revealed firstly the mitochondrial genome sequence of the *S. hainanensis*. This mitochondrial genome was a circular DNA molecule of 59,508 bp, including 24 transfer RNA genes, 3 ribosomal RNA genes, and 31 protein-coding genes. The GC content of the genome was 35.4%. The phylogenetic tree suggested that *S. hainandiae* was a sister to the OUU clade within the class Ulvophyceae. The mitochondrial genome structure and gene content of *S. hainanensis* supported that *S. hainanensis* was a new unidentified green alga in Ulvophyceae.

## 1. Introduction

*Symbiochlorum hainanensis* Gong et al. (2018), a unicellular green alga, belongs to Ulvophyceae, Chlorophyta, and was firstly identified and described as a new species by Gong et al. (2018). This species is found in various corals in the tropical coral reef areas of the South China Sea. *S. hainanensis* is a thermo-tolerant alga, which may play vital roles in coral reefs, especially coral-algae symbiont (Gong et al. 2020). We also find similar phenomenon that *S. hainanensis* can maintain rapid growth at high temperature. Mitochondrial genome is known to play important roles in the studies of population genetics, phylogeny, and evolution. In the present study, we analyzed the complete mitochondrial genome of *S. hainanensis*, and determined its phylogenetic position.

## 2. Materials

**Figure 1.**
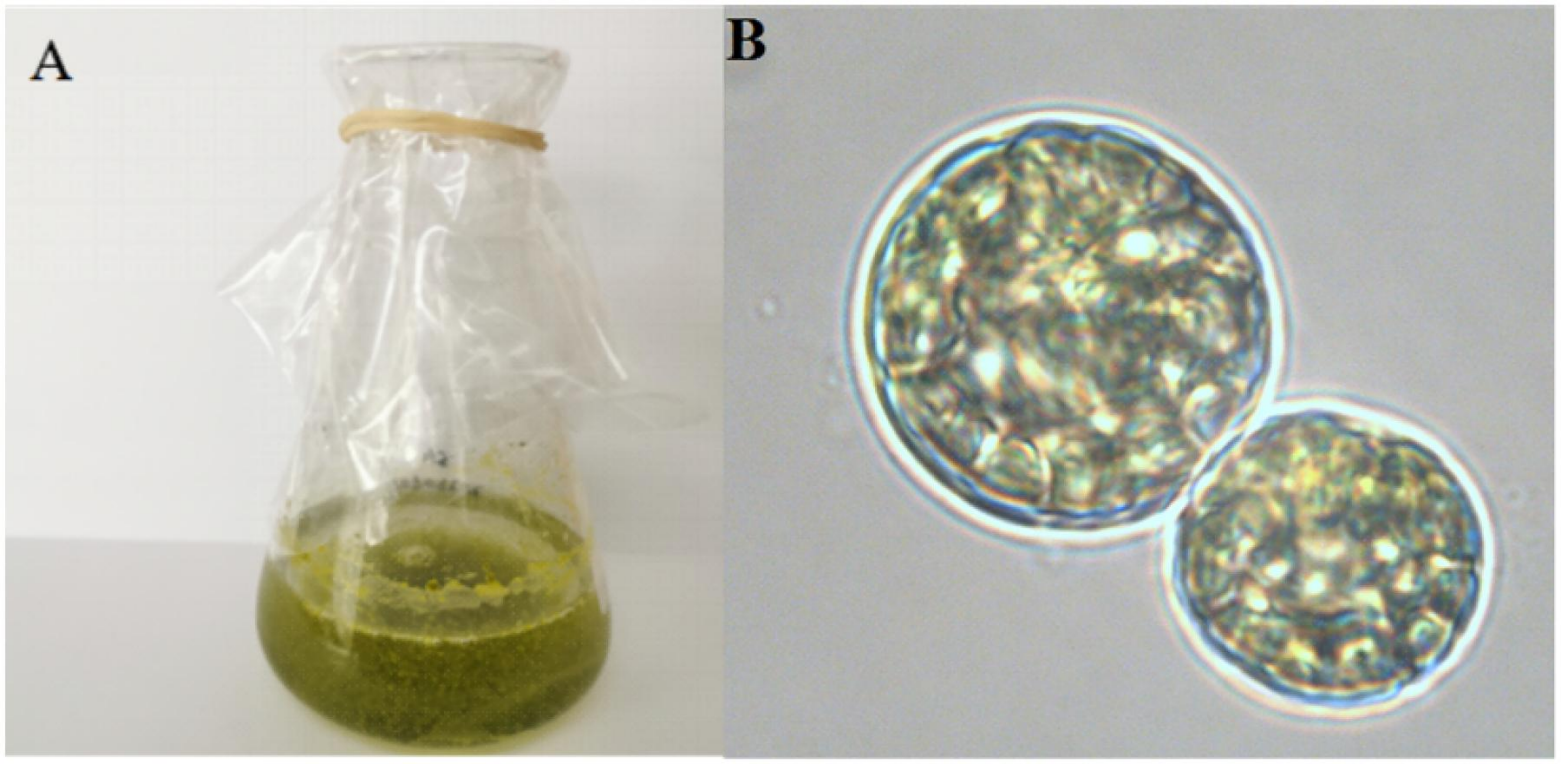
The morphology of *S. hainanensis* (A) in flasks and (B) under a microscope. Photograph was taken by Fangfang Yang.

*S. hainanensis* was collected from coral *P. damicornis* in the coast of Sanya City, China (18°21′ N and 109°47′ E), and deposited at the laboratory of South China Sea Institute of Oceanology, Chinese Academy of Sciences (Fangfang Yang, ycuyang@scsio.ac.cn) under the voucher number SY20210606 (Figure 1).

## 3. Methods

The genomic DNA was extracted using the CTAB protocol (Doyle 1987). A DNA library was prepared using TruSeq Nano DNA Kit and sequenced on Illumina Novaseq 6000 Sequencing platform (Illumina, CA, USA). The genomic DNA was generated about 4.75 G raw reads, and 4.69 G clean data was obtained from the raw data after quality control processing. The circularized genome was de novo assembled from the high-quality paired-end reads by using the SPAdes 3.14.1 program based on the K-mer size of 10^5^ (Bankevich et al. 2012). The mitochondrial genomes were annotated using MFannot and Mitofy (Bernt et al. 2013). The average depth was over 200X (Figure S1). The mitochondrial genome map were drawn using the CPGview program (http://www.1kmpg.cn/cpgview/) (Liu et al. 2023). Finally, the mitochondrial genome data was uploaded NCBI with accession number ON897766. The 13 common protein-coding genes in each complete mitochondrial genome of 9 species were aligned with the genes in *S. hainanensis* using MAFFT 7.037 (Katoh and Standley, 2016) with the FFT-NS-2 strategy. Then, model-finder var 1.6 was run to select the best-fit model and the cpREV + F + I + G4 model was chosen (Kalyaanamoorthy et al. 2017). Finally, iqtree 2.0 was used to construct a phylogenetic tree with 1000 bootstraps based on the ML method (Minh et al. 2020).

## 4. Results

**Figure 2.**
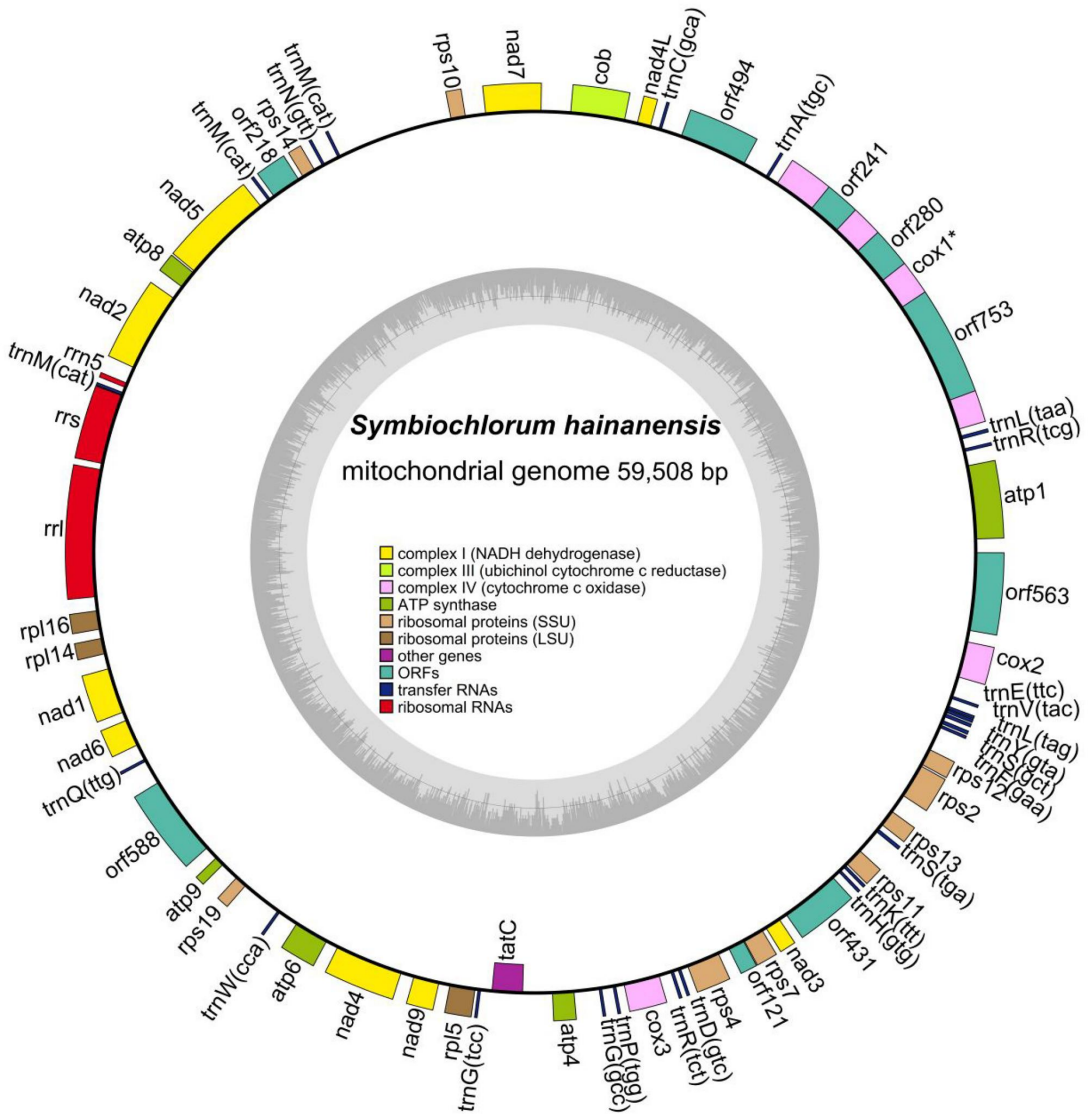
The complete mitochondrial genome map of *S. hainanensis*. Arrangement of 58 genes represented in the map, including 31 protein-coding genes, 24 transfer RNA genes, and 3 ribosomal RNA genes.

**Figure 3.**
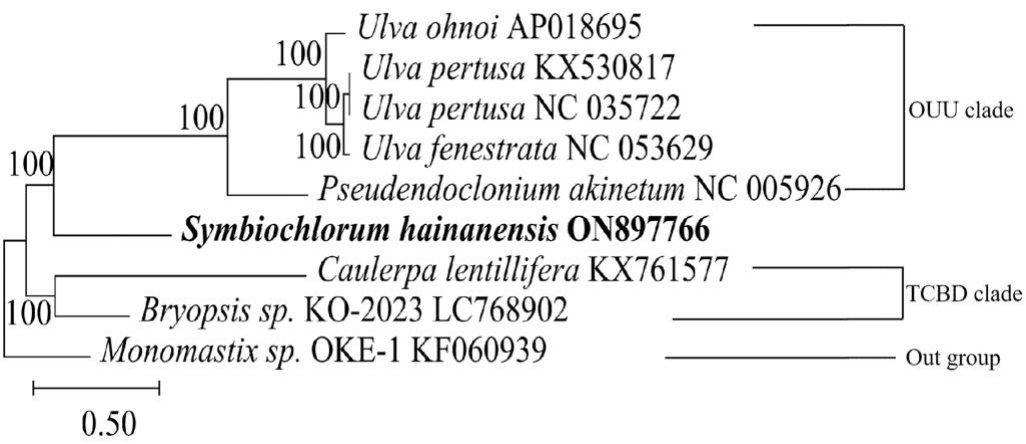
Neighbor-joining phylogenetic tree of *S. hainanensis* and 8 other closely related species based on the complete mitochondrial genomes. The following sequences were used: *Symbiochlorum hainanensis* (ON897766), *Ulva fenestrata* (NC_053629), *Ulva ohnoi* (AP018695), *bryopsis* sp. KO-2023 (LC768902), *Ulva pertusa* (NC_035722), *pseudendoclonium akinetum*(NC_005926), *Ulva pertusa* (KX530817), *caulerpa lentillifera* (KX761577), and *monomastix* sp. OKE-1(KF060939).

This mitochondrial genome was a circular DNA molecule of 59,508 bp, including 24 transfer RNA genes, 3 ribosomal RNA genes, and 31 protein-coding genes (PCGs) (Figure 2). The complete mitochondrial genome of *S. hainanensis* was in the range of known Ulvophyceae mitogenome sizes (55,814–88,416 bp) (Pombert et al. 2004; Liu et al. 2022). The overall bases were composed of 31.7% A, 32.9% T, 16.6% C, and 18.8% G. Most of the PCGs initiated with the standard ATG except for rps10 ACG with the start codon. Four types of stop codons were TAG (*cob*, *atp8*, *rpl16*, *nad1*, *nad4*), TGA (*rpl5*, *tatC rps4*, *rps13*, *rps2*), GTT (*nad7*), and the remaining genes with the TAA codon. There are no difficult-to-annotate trans-splicing genes in the mitochondrial genome, but there is a cis-splicing gene called COX1 (Figure S2). Phylogenetic analysis showed that *S. hainanensis* was sister to OUU clade and TCBD clade within the class Ulvophyceae (Fang et al. 2017) (Figure 3).

## 5. Discussion and conclusion

In this study, the complete mitochondrial genome of *S. hainanensis* was sequenced, assembled, and annotated for the first time. The mitochondrial genome of *S. hainanensis* was 59,508 bp in length, and it contained 31 protein-coding genes, 24 transfer RNA genes, 3 ribosomal RNA genes. The genomic structure of *S. hainanensis* was similar to the OUU clade (Zhou et al. 2016). This study will enhance the evolution and application study of the class Ulvophyceae.

## Supplementary Material

Supplemental Material

## Data Availability

The genome sequence data that support the findings of this study are openly available in GenBank of NCBI at (https://www.ncbi.nlm.nih.gov/) under the accession no. ON897766. The associated BioProject, SRA, and Bio-Sample numbers are PRJNA844566, SRR19521163, and SAMN28834016, respectively.
