# Supplementary material for: Complete mitochondrial genome and phylogenetic analysis of the marine microalga Symbiochlorum hainanensis (Ulvophyceae, Chlorophyta)
Source: Mitochondrial DNA B Resour. 2023 Dec 18;8(12):1377–80. doi: 10.1080/23802359.2023.2290353 (PMC10732211; doi:10.1080/23802359.2023.2290353)
Supplement: Supplemental Material [file TMDN_A_2290353_SM5820.doc]

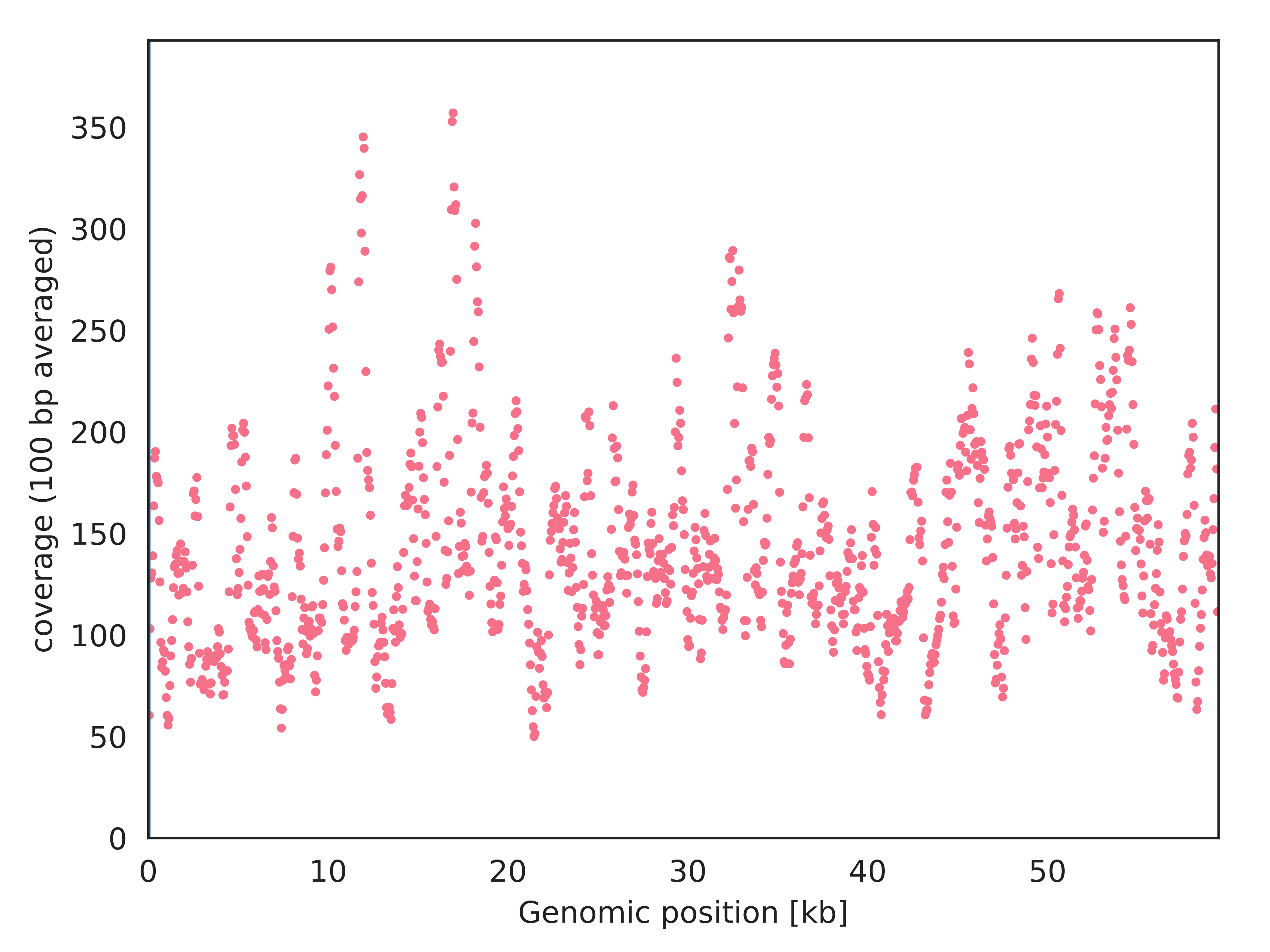


Figure S1 Coverage depth figure of the *Symbiochlorum hainanensis*.

*
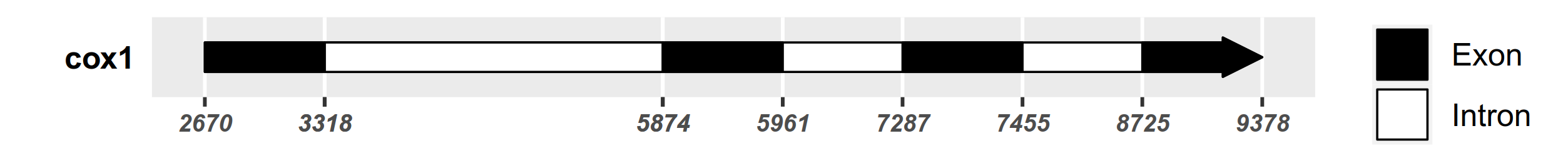
*

Figure S2 Schematic map of the cis-splicing genes in the *Symbiochlorum hainanensis* complete mitochondrial genome.
